# Evaluation of total alloplastic temporo-mandibular joint replacement with two different types of prostheses: A three-year prospective study

**DOI:** 10.4317/medoral.21189

**Published:** 2016-07-31

**Authors:** Luis-Miguel Gonzalez-Perez, Borja Gonzalez-Perez-Somarriba, Gabriel Centeno, Carpóforo Vallellano, Jose-Francisco Montes-Carmona

**Affiliations:** 1 Department of Oral and Maxillofacial Surgery, Virgen del Rocio University Hospital. Seville. Spain; 2 Department of Mechanical and Manufacturing Engineering, Engineering School. University of Seville. Spain

## Abstract

**Background:**

Temporo-Mandibular Joint (TMJ) replacement has been used clinically for years. The objective of this study was to evaluate outcomes achieved in patients with two different categories of TMJ prostheses.

**Material and Methods:**

All patients who had a TMJ replacement (TMJR) implanted during the study period from 2006 through 2012 were included in this 3-year prospective study. All procedures were performed using the Biomet Microfixation TMJ Replacement System, and all involved replacing both the skull base component (glenoid fossa) and the mandibular condyle.

**Results:**

Fifty-seven patients (38 females and 19 males), involving 75 TMJs with severe disease requiring reconstruction (39 unilateral, 18 bilateral) were operated on consecutively, and 68 stock prostheses and 7 custom-made prostheses were implanted. The mean age at surgery was 52.6±11.5 years in the stock group and 51.8±11.7 years in the custom-made group. In the stock group, after three years of TMJR, results showed a reduction in pain intensity from 6.4±1.4 to 1.6±1.2 (*p*<0.001), and an improvement in jaw opening from 2.7±0.9 cm to 4.2±0.7 cm (*p*<0.001). In the custom-made group, after three years of TMJR, results showed a reduction in pain intensity from 6.0±1.6 to 2.2±0.4 (*p*<0.001), and an improvement in jaw opening from 1.5±0.5 cm to 4.3±0.6 cm (*p*<0.001). No statistically significant differences between two groups were detected.

**Conclusions:**

The results of this three-year prospective study support the surgical placement of TMJ prostheses (stock prosthetic, and custom-made systems), and show that the approach is efficacious and safe, reduces pain, and improves maximum mouth opening movement, with few complications. As such, TMJR represents a viable technique and a stable long-term solution for cranio-mandibular reconstruction in patients with irreversible end-stage TMJ disease. Comparing stock and custom-made groups, no statistically significant differences were detected with respect to pain intensity reduction and maximum mouth opening improvement.

**Key words:**Temporo-mandibular joint, temporo-mandibular joint replacement, prosthesis, biomaterials, biomedical engineering, computer-aided design and manufacturing.

## Introduction

Prostheses are artificial devices used to replace body parts due to degenerative diseases, accident trauma or tumours (1). The history of cranio-maxillofacial reconstruction with prostheses is interspersed with multiple failures due to inappropriate design, lack of attention to biomechanical principles, and a lack of familiarity with what has already been documented in the literature on biomaterials (2). Prosthetic joint replacements provide a biomechanical solution to advanced disease. In its present form, Cranio-Mandibular Joint Replacement has been used clinically for over 20 years and today remains one of the most successful applications for reconstruction of an irreversibly damaged tissue. The number of prostheses being implanted is increasing at a significant rate, with the success of the surgical procedure, the increased longevity of the population, the demand for increased quality of life and more active lifestyles, and the earlier diagnosis of diseases meaning that Temporo-Mandibular Joint Replacement (TMJR) is now undertaken across a wider age-range of patients. This has placed increased demands on both the design and the performance of prostheses (3,4).

The physical environment into which the prosthesis is implanted is extremely challenging. Not only do biomechanical characteristics have to be contemplated, but also the fact that the tissue surrounding the prosthetic components remains living means that the prosthetic device interface and tissue environment can continually change with time. These changes are not only related to the natural ageing of the patient, but can also occur in response to the func-tion and properties of the prosthetic device itself. This complex biological, biomechanical, biomaterial interaction can determine the lifetime of the prosthesis. Over the years it has proven very difficult to predict preclinically many of these interactions, and it is only as a result of clinical experience and research that particular clinical success and failure scenarios have emerged. This has resulted in more rigorous and demanding requirements for prosthetic replacement designs and materials, although the ultimate test is the long-term clinical follow-up (5,6).

Progress in medical imaging and continued advances in computer processing power for three-dimensional data acquisition of patient parameters and subsequent image processing make it possible for surgeons to diagnose their patients, more accurately plan and simulate model prostheses, and carry out appropriate treatments. The aim of this study is to evaluate procedures, complications associated, and the differences in pain levels and in maximal incisal opening in patients treated with stock and custom TMJ prostheses. This work has been carried out within the framework of a collaborative study between the School of Engineering and one of the main teaching hospitals of the University of Seville.

## Material and Methods

- Subjects

**Figure 1:**
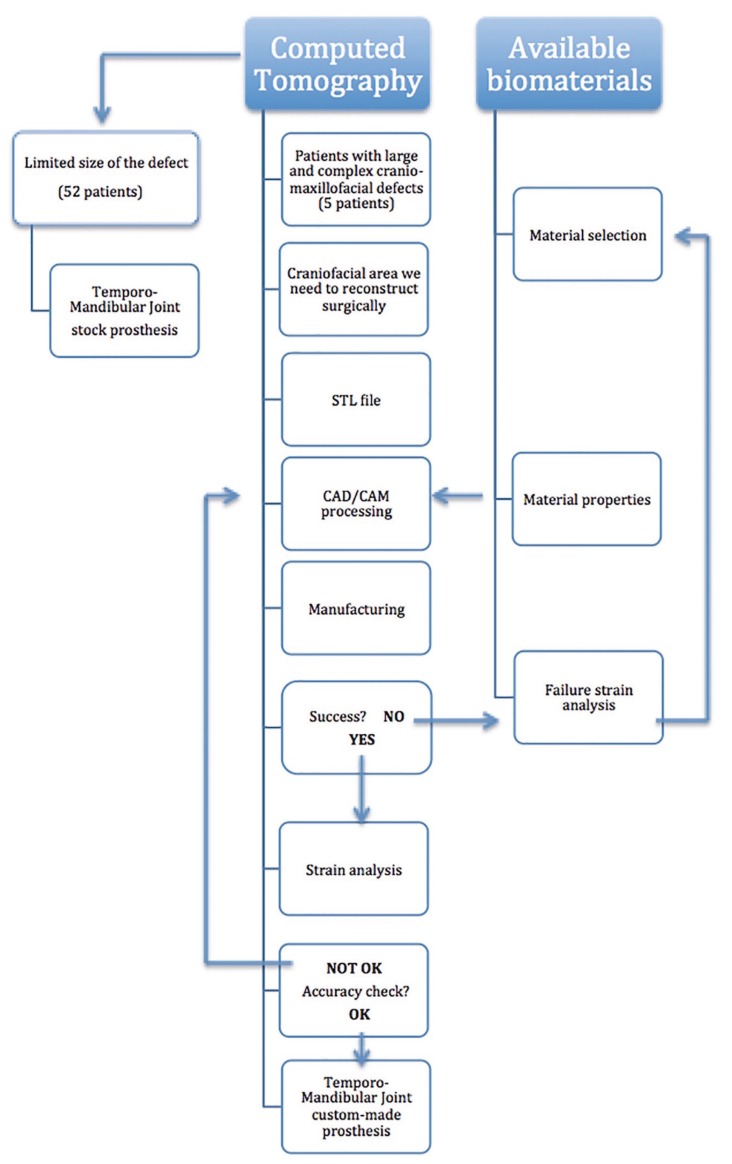
Surgical workflow of methodology used for cranio-mandibular prosthesis.

All the patients who had a Cranio-Mandibular Joint Replacement implanted during the study period from January 2006 through December 2012 and who were operated on were included in this 3-year prospective study. All procedures were performed using the Biomet Microfixation TMJ Replacement System® (stock prosthetic system, and custom-made system), and all involved replacing both the skull base component (glenoid fossa) and the mandibular condyle with a specific workflow (Fig. 1,  Table 1). The glenoid fossa and mandibular components were available in three different sizes in the stock prosthetic system, and with patient specific imaging data in the custom-made prostheses. The mandibular component of the TMJR was manufactured from cobalt-chromium-molybdenum (Co-Cr-Mb) alloy with a roughened titanium plasma coating on the host bone side of the ramal plate for increased bony integration. The specific cobalt-based alloy used was ASTM (American Society for Testing and Materials) type F799. The fossa prosthesis was made of ultra-high-molecular-weight-polyethylene (UHMWPE). All parts of TMJ prostheses were implanted in patients under general anesthesia. In the stock prosthetic devices, templates were used intraoperatively to determine the fit and only then was the final cranio-maxillofacial prosthesis inserted. The accuracy of custom-made implants makes the use of templates unnecessary. Screws used in the procedure were made of 6Al/4V titanium alloy. The following inclusion diagnostic criteria were assessed: A history of persistent pain (meaning pain present at least 8 hours/day≥15 days per month) in the TMJ area accompanied by functional impairment (jaw opening<40mm or lateral motion<5mm or protrusive range<5mm) after failure of other non-surgical and surgical therapies, and imaging evidence consistent with advanced TMJ disease (panoramic radiography, computed tomography and/or magnetic resonance imaging) of more than one year’s duration. Previous non-surgical treatments included a combination of pharmacological treatments, splint therapy, and physiotherapy from a multidisciplinary approach. Previous surgical therapies included arthrocentesis, arthroscopic surgery, remodeling of the joint surface, removal of the articular disc, and partial replacement of TMJ components. Subjects were excluded if they presented with one or more of the following conditions: insufficient quantity or quality of bone to support the cranio-maxillofacial replacement, severe hyperfunctional habits, active infectious disease, or incapacity to follow postoperative care instructions. The Research and Clinical Ethics Committee of the lead author’s institution approved the study (2013PI/119). Declaration of Helsinki guidelines were followed. Before inclusion, all patients signed an informed consent form.

**Table 1:**
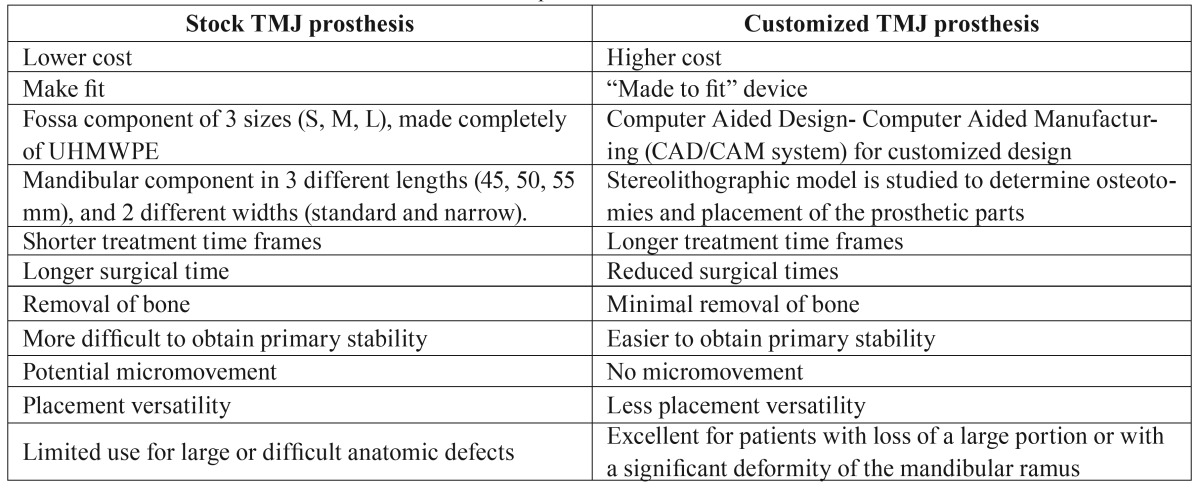
Differences between stock and custom-made TMJ prostheses.

- Study Design

Evaluations were carried out on preoperative day 1, and at months 3, 6, 12, 24, and 36 following the TMJR. Data were collected at each visit by the same observer who had not been involved in any of the surgical procedures.

- Measures

Pain intensity changes (preoperative vs. current) were measured using a visual analog scale (VAS 10 cm), ranging from 0 to 10 with higher scores indicating more severe pain. Jaw opening was evaluated with a Therabite® scale between the incisal edges of the upper and lower central incisors. Panoramic radiographs and computed tomography studies were obtained immediately postoperatively and at follow-up visits for evaluation and comparison. Surgical morbidity and implant survival were documented. The main parameters to assess the effectiveness of the treatment were: 1) pain at rest and upon mastication as measured by the VAS, and 2) range of mandibular movements associated with opening of the mouth, measured with a Therabite® ruler. Continuous normally distributed data were expressed as the mean ± standard deviation (SD) and compared with the Student´s t-test; all other data were expressed as percentages. Signs that were evaluated as indicators of the effectiveness of the TMJR were: significant reduction in TMJ pain at rest and with mastication, and recovery of normal ranges of mouth opening movements.

- Statistical Analysis

Data were analyzed with statistical software (IBM SPSS Statistics 19.0). The Kolmogorov-Smirnov test for normality was used for quantitative variables, which were expressed as average ± SD or as the 25th-75th percentile (P25-P75, interquartile range). Values of *p*<0.05 were considered to indicate statistical significance.

## Results

Fifty-seven patients (38 females (66.7%) and 19 males (33.3%), involving 75 TMJs with severe disease requiring reconstruction (39 unilateral, 18 bilateral) were operated on consecutively. Sixty-eight stock total joint prostheses (16 bilateral (30.8% of this group of patients) and 36 unilateral (69.2%): 20 right TMJ/ 16 left TMJ) and 7 custom total joint prostheses (2 bilateral (40% of this group patients), and 3 unilateral (60%): 2 right TMJs/ 1 left TMJ) were implanted during the study period. The mean age at surgery was 52.6 ± 11.5 years (range, 29-74 years) in the stock group and 51.8 ± 11.7 years (range, 39-64 years) in the custom-made group.

**Figure 2:**
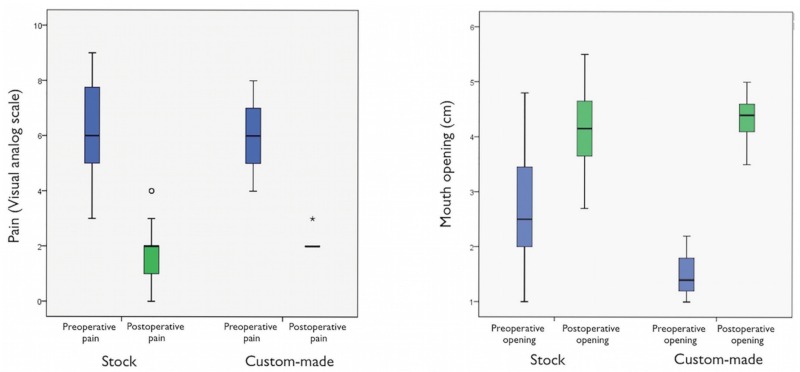
The results of our study show, for a three-year follow-up after stock TMJR, a significant mean reduction in pain intensity, and a significant mean improvement in jaw opening. In the custom-made group, pain intensity was significantly decreased postoperatively, and mouth opening significantly increased postoperatively. No statistically significant differences between two groups were detected.

**Figure 3:**
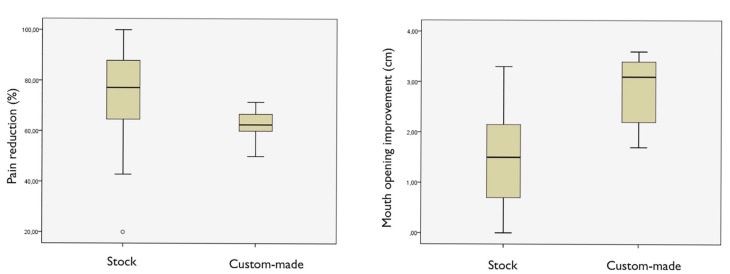
As the TMJR is a complex, interactive biomechanical and biological system; long-term clinical results remain the ultimate test of efficacy and functionality. Three years after cranio-mandibular replacement, results showed a reduction in pain intensity, and an improvement in jaw opening from the preoperative average.

In the stock group, after three years of cranio-mandibular replacement, results showed a reduction in pain intensity from an average pain score of 6.4 ± 1.4 to 1.6 ± 1.2 (*p*<0.001), and an improvement in jaw opening from the preoperative average of 2.7 ± 0.9 cm to 4.2 ± 0.7 cm (*p*<0.001) ( Table 2, Fig. 2). Patients in this stock group had a pain reduction of 76.5 ± 18.5 points (Fig. 3). The mean follow-up period from initial TMJ symptoms to TMJR surgery was 5 years (range: 1-8 years). The average number of previous TMJ surgeries, including arthrocentesis and arthroscopy, was 2 (range: 0-10) for each joint. Nine patients (17.3%) had an obvious history of mandibular trauma. Twenty-one patients (40.3%) had a TMJ tumoural pathology (one TMJ in 18 patients / both TMJs in three patients), 15 patients (28.8%) had severe degenerative osteoarthritis (8 bilateral/ 7 unilateral), nine patients (17.3%) had total fibrous/bony TMJ ankylosis (6 unilateral/ 3 bilateral), six patients (11.5%) had post-traumatic sequels (5 unilateral/ 1 bilateral), and one patient (1.9%) had unilateral severe rheumatoid arthritis. All diagnoses were confirmed by histopathological examination.

**Table 2:**
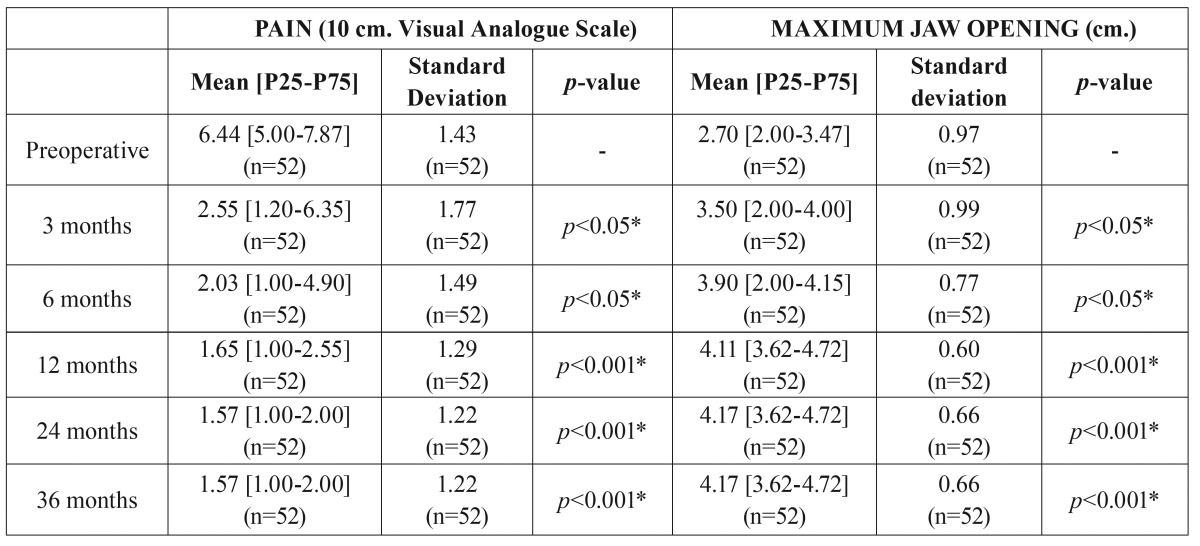
Outcome measures for all patients (stock group; n=52) during the study period.

In terms of custom-made prostheses, pain intensity was significantly decreased postoperatively and was sustained for three years. The pain intensity in the custom-made group was 6.0 ± 1.6 preoperatively, whereas at 3 years postoperatively it was 2.2 ± 0.4, demonstrating a statistically significant decrease in pain (*p*<0.001) ( Table 3, Fig. 2). We observed an average pain reduction of 62.1 ± 8.5 percentage points in this group (Fig. 3). Furthermore, mouth opening significantly increased postoperatively compared with preoperative measures. The preoperative mean was 1.5 ± 0.5 cm, and at 3 years the postoperative average was 4.3 ± 0.6 cm, showing a statistically significant increase in maximum mouth opening (*p*<0.001) ( Table 3, Fig. 2). The mean follow-up period from initial TMJ symptoms to TMJR surgery was 6 years (range: 2-12 years). The average number of previous TMJ surgeries, including arthrocentesis and arthroscopy, was 3 (range: 2-6) for each joint. Three patients (60%) had a history of post-traumatic sequels (1 unilateral/ 2 bilateral), and two patients (40%) had unilateral total bony TMJ ankylosis. All diagnoses were confirmed by histopathological analysis. None of the surgical procedures were carried out in patients who had good mandibular motion. All of the TMJ replacements were carried out in patients that had end-stage joint disorders as a result of which none of the TMJ components can be salvaged. Our study included 21 patients with TMJ tumours in the stock group where the condyle was severely deformed and its radical resection was the only guarantee that it will not recur; although in 8 of these cases mouth opening can be considered within the normal range of mandibular mobility (≥4 cm), all of them were patients with a history of progressive mandibular deviation, open bite on the ipsilateral side and posterior crossbite, and diagnostic imaging with evidence of catastrophic changes to the TMJ. After TMJR, all patients were followed up for at least three years. No patient’s symptoms had worsened postoperatively. Comparing stock and custom-made groups, no statistically significant differences were detected with respect to pain intensity reduction and maximum mouth opening improvement compared, although conclusions about this data should be considered carefully given the small number of patients in the custom-made group (Figs. 2,3).

**Table 3:**
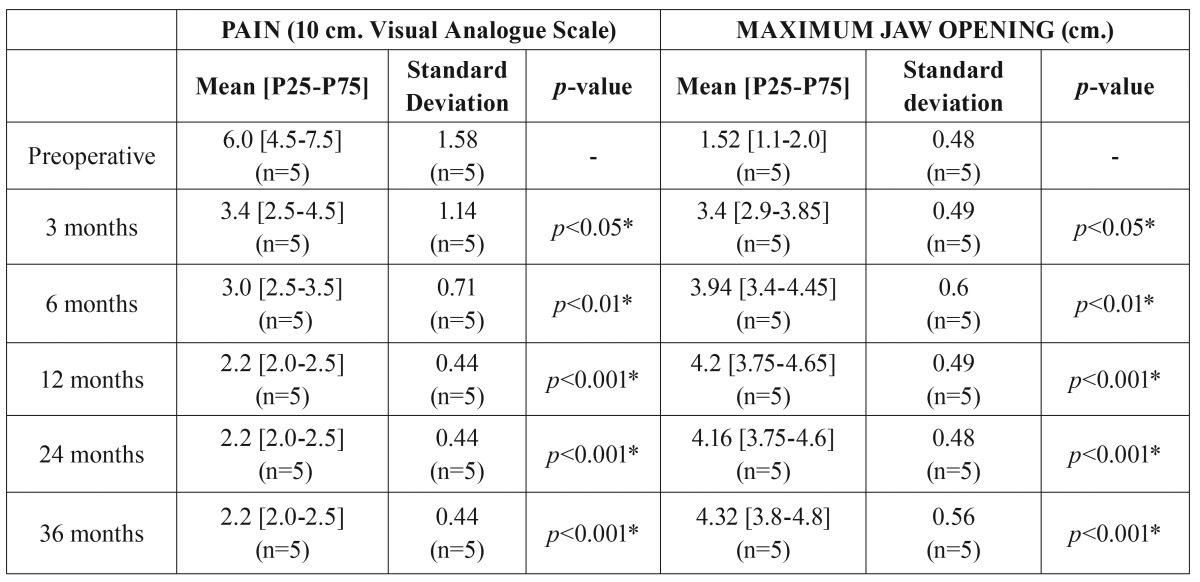
Outcome measures for all patients (custom-made group; n=5) during the study period.

Complications after stock total TMJR can be summarized as follows: three patients had a numb lip which resolved spontaneously; three patients showed a tendency towards an incorrect occlusal position one year after cranio-maxillofacial replacement, indicating instability of the prosthesis (one patient with open bite, one with a Class II malocclusion, and one with a relapsing dislocation); two patients had temporary weakness of the temporal branch of the facial nerve; one patient showed severe hypersensitivity to the cobalt- chromium alloy, and one patient had heterotopic bone formations. In two cases, one involving an anterior open bite deformity and the other with a class II malocclusion (which was the result of loosening of screws), the implants were removed and two new total replacements were fitted. The patient with dislocation of the TMJ prosthesis was treated by elastic intermaxillary fixation for 10 days, after which there were no further episodes of dislocation. The case of severe hypersensitivity to the cobalt-chromium alloy was also cause for re-intervention, with the mandibular prosthetic component replaced with a titanium-alloy component. There was a progressive decrease in jaw range of motion without worsening of pain in another patient with bilateral cranio-maxillofacial replacement. The predominant finding in the computed tomographic image was a voluminous calcified mass surrounding the TMJR as evidence of heterotopic calcifications. This patient was re-operated to bilaterally remove the heterotopic bone formations. The prostheses were not removed in this case, but debridement was performed around them, and autologous fat grafts from the abdominal suprapubic area were placed into the joint space and around the prosthesis to prevent fibrosis and heterotopic bone formation. No cases of UHMWPE particulation-related osteolysis were identified. No patient reported 7th nerve dysfunction after one year. Only one patient had a postoperative complication in the custom-made group with temporary weakness of the temporal branch of the facial nerve.

## Discussion

The design and development of TMJR is a highly interdisciplinary activity, calling for an understanding of Material Science and Engineering, detailed knowledge of the TMJ anatomy, and surgical experience (1,5-7). The TMJ is one of the most active joints in the human body, resulting in TMJ diseases being a common problem. The resection and replacement of the diseased TMJ is, however, usually reserved for patients with irreversible end-stage disorders (8-10). TMJR has been one of the major success stories in TMJ surgery in recent years, with clinical success, long-term results, and increased expectation and lifetimes of patients driving the need for improved materials, load-bearing surfaces and designs. This unique “ball and socket” prosthesis is capable of rotational and translational movements, and allows for more than 2000 hinge and sliding motions per day in activities such as eating, speaking, and swallowing. The choice of appropriate materials and designs begins with an understanding of engineering concepts and desirable prosthetic implant properties (density, elastic modulus, stress shielding, notch sensitivity, tribocorrosion) (1,11). The biomaterials from which prosthetic implants are made must be biocompatible, and any wear particles produced must be compatible with the body and not cause adverse biological reactions. The TMJR must be compatible with a range of different patient anatomies and geometries and typically a range of different sizes is necessary. Similarly, the bone quality of patients varies considerably and the methods of fixation must be able to accommodate different bone interface conditions (12).

In terms of considering if the implanted material may invoke a tissue response, the materials used remain basically the same as ever, namely, metal alloys, and polymers (mostly polyethylene). Bioactive coatings and particulate materials constitute the last broad category to which the body reacts in cranio-maxillofacial replacement. Employing the most advantageous physical characteristics of biocompatible materials is an essential consideration in the design and manufacture of any TMJR device (13). In our experience, Co-Cr-Mb alloy, with its relatively high carbon content, contributes to its strength, polishability, and biocompatibility. Its excellent wear characteristics when articulated against an UHMWPE material presently make it the standard for the non-moveable articulating surface of most orthopaedic total joint replacement devices. Cobalt-based alloys were initially used as an orthopedic biomaterial because they were more corrosion-resistant than stainless steel. However, cast Co-Cr, often employed in the manufacture of stock cranio-maxillofacial replacement devices, is biomechanically inferior to any wrought alloy employed in custom-made prostheses. Metallurgical flaws such as inclusions and porosity found in cast Co-Cr components have been associated with the fatigue failure of metal-on-metal prostheses. These flaws may also lead to the long-term failure of Co-Cr TMJR components, resulting in noxious metallic debris (metalosis) found in adjacent tissues. UHMWPE is a linear unbranched polyethylene chain with a molecular weight of more than one million; testing of this material for over one decade in TMJ prostheses has led to the conclusion that UHMWPE has excellent wear and fatigue resistance for a polymeric material. Methods of fixation of the cranio-maxillofacial replacement to the bone have progressed through coatings that allow bone ingrowth (hydroxyapatite) to bioactive coatings for so-called osteointegration (titanium plasma coating) (13).

Two categories of TMJR devices have been approved for implantation: stock-devices which the surgeon must fit at implantation, as occurred in 52 of our cases, and patient-fitted or custom devices which are made specifically for each patient, as occurred in 5 of our cases ( Table 1, Fig. 1). The process associated with fabricating a custom-made cranio-maxillofacial replacement on the basis of a model generated from a computed-tomography scan is often time consuming and expensive. The complexity of the anatomy of the TMJ presents problems with its reconstruction, with many of the movements of the normal joint not having been reproduced in artificial joints made available to the present time. As the TMJR is a complex, interactive biomechanical and biological system, long-term clinical results remain the ultimate test of efficacy and functionality. The results of our study show, for a three-year follow-up after stock TMJR, a significant mean reduction in pain intensity, and a significant mean improvement in jaw opening. In the custom-made prosthesis group, pain intensity was also significantly decreased postoperatively, and mouth opening was significantly increased. All procedures in our study were performed using the Biomet Microfixation TMJ Replacement System® (68 stock prostheses, and 7 custom-made prostheses), but no statistically significant differences between two groups were detected with respect to pain reduction and mouth opening improvement, although the data should be considered with care due to the small number of patients in the custom-made group (Figs. 2,3).

Our study also found that patients with a poorer functional status prior to treatment obtained the best final outcomes. The results in our series indicated implant failure in just 3 of 68 implants in the stock Temporo-Mandibular Joint Replacement group (4%); in two of these cases there was a tendency towards an incorrect occlusal position one year after cranio-maxillofacial replacement, indicating instability of the prosthesis, while another patient showed severe hypersensitivity to the cobalt-chromium alloy. It should be noted that one bilateral TMJR patient showed a progressive decrease in range of motion due to heterotopic bone formation, but in this case there was no need to replace the prosthesis. Radiological study is useful to exclude other pathological processes such as marked osteolysis or a fracture. There are no specific features relating to infection in and around prosthetic joints. Ordinary radiographs are not sufficiently sensitive or specific, while computed tomography and magnetic resonance imaging are both limited by artifacts induced by the implanted hardware. Recent developments in metal artifact reduction techniques in magnetic resonance have, to a significant extent, been stimulated by the occurrence of soft tissue complications associated with modern cranio-maxillofacial reconstruction, and may require additional input from engineers. Inversion recovery sequences can be readily modified to ameliorate metal artifact, and newer multispectral imaging techniques promise significant improvements in magnetic resonance imaging of soft tissues around prostheses. This has become particularly important with modern prostheses in which conventional radiographs are often normal despite extensive soft tissue disease (14).

The relative novelty of the modern cranio-maxillofacial replacement limits the availability of long-term data regarding material wear, stability, and implant failure. The longevity of the TMJR thus remains unknown. It has been demonstrated that the use of appropriate biomaterials and design parameters can decrease material wear and increase the longevity of TMJR devices (2,6). In our study, there were no cases of UHMWPE wear-related osteolysis, but two patients had instability of the prosthesis as a result of loosening of the screws. Although the anatomical fit of the fossa and mandibular components enhances the stability of TMJR, there is no argument to support the fact that because a custom prosthesis is based on an exact fit to the bone it will likely offer greater longevity. Opponents of the stock TMJR system state that such prostheses have an inferior fit owing to repeated trying-in of prosthetic components to determine the closest fit, but estimating the ideal size prior to the operation by simply overlaying the components of the stock joints on plain radiographs can drastically decrease this, as we did for our patients in the stock prosthesis group. The data analysis from our study also revealed that the need for TMJR involves a relatively younger patient population with a mean age at surgery in the stock group of 52.6 ± 11.5 years (range, 29-74 years) and 51.8 ± 11.7 years (range, 39-64 years) in the custom-made group. As 38 % of the patients were under the age of 50 years at the time of surgery, this means that the TMJR must have a long lifetime because once the prosthesis is implanted there is no way to return to the previous anatomy. The longevity of cranio-maxillofacial replacement devices is based on the proper indication for its use, the properties and biocompatibility of the materials used, the correct placement and stability of the prosthesis in situ, the patient’s biological acceptance of the device, and the capacity of the patient to understand the limitations involved with having a prosthesis in place. Our prosthetic implantations were designed with all of these factors in mind (15).

The prosthetic materials must be anatomical in shape, be securely fixed to the surrounding bone, and remain securely fixed throughout the patient’s lifetime ( Table 4) (16); two of our cranio-maxillofacial replacement were explanted due to malocclusion, which was a result of the loosening of screws. Increased loading post-surgery occurs until the TMJRs, muscles, soft tissues and occlusion reach a state of equilibrium and adaptation to the new position, which could take several months. As such, we consider the initiation of postoperative physiotherapy to be very important, as was done with our patients.

**Table 4:**
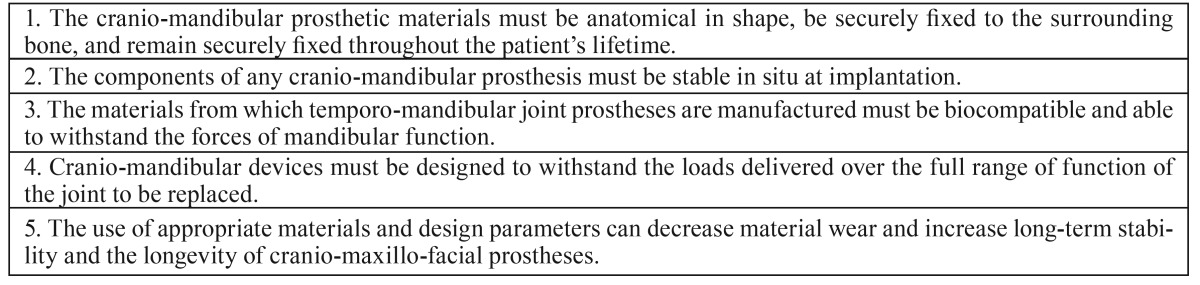
Established criteria for successful Cranio-Mandibular Replacement.

The main problems associated with TMJR are related to wear at the articular surfaces, foreign body reaction, and mobility of the implant with displacement and implant fracture caused by the use of inappropriate alloplastic materials (11). A number of different prostheses were available for this procedure, including TMJ Implants, TMJ Concepts, and the Biomet Microfixation TMJ Replacement System; nevertheless, since early 2006 nearly all TMJ prostheses implanted in our department have had a UHMWPE glenoid fossa cup. To this end, while metal-on-metal stock TMJRs were introduced a long time ago and have been used in our unit over the last 15 years, with similar outcomes to the UHMWPE-on-metal prosthesis, the numbers used are too low to enable a comparative analysis to be performed. The debate in the literature relating to the efficacy of total joint replacement appears to indicate that joints made from cobalt-chromium alloy articulating with UMWPE fulfill the requirements orthopaedic surgeons have used for artificial joint replacements in the hip, knee and shoulder (6). Studies by other authors show that TMJR has been successfully employed in the 20 years they have been following their patients (1,3,4). Hypersensitivity can also present a problem, with nickel, cobalt and chromium being the most common sensitizing agents. This hypersensitivity may be the trigger for unfavourable outcomes with total joint surgery, as occurred in one of our patients (5). For this reason, a metal allergy test patch has been included in the preoperative studies for TMJR patients at our institution.

One deficiency in planning cranio-maxillofacial surgery is the inability to predictably produce complex temporo-mandibular contours using commercially available stock TMJR devices, which are supplied as generic sizes and shapes designed on the basis of the average patient (5,7,16). In the most complex and difficult cases, the surgeon may spend considerable time during surgery shaping the cranio-mandibular replacement to fit the contour of the patient’s bone, and these repeated manipulations to adapt them to difficult anatomical confines might make the prosthesis susceptible to fatigue fractures (16). One solution to this problem is to use computer-guided surgical planning technologies to produce a passive fitting cranio-maxillofacial prosthesis designed for specific anatomical needs of patients. Progress in medical imaging and continued advances in computer-processing power for three-dimensional data acquisition of patient parameters and subsequent image processing make it possible for clinicians to diagnose, more accurately plan, simulate and treat end-stage TMJ patients. To the present time, the most common use of additive manufacturing has been the fabrication of patient specific skull models, which are fabricated for preoperative planning using patient-specific imaging data in Digital Imaging and Communications in Medicine (DICOM) files, which are then converted into stereolithography (SLT) files, the standard manufacturing format used to print patient specific skull models. The use of such three-dimensional medical models helps surgeons to plan, simulate the planned operation and manually pre-shape commercially available cranio-maxillofacial replacement devices. Recent developments in the area of additive manufacturing allow the prefabrication of patient specific, custom-made prostheses using the patient’s DICOM data. The advantages of rapid prototyping in designing and manufacturing customized cranio-maxillofacial prostheses are that they do not require intraoperative modifications and offer improved passive fitting (17).

To conclude, the results of this three-year prospective study support the surgical placement of TMJ prostheses (stock prosthetic, and custom-made systems), and show that the approach is efficacious and safe, reduces pain, and improves maximum mouth opening movement, with few complications. As such, TMJR represents a viable technique and a stable long-term solution for cranio-mandibular reconstruction in patients with irreversible end-stage TMJ disease. Improvements persisted for three years following completion of the surgical treatment. Comparing stock and custom-made groups, no statistically significant differences were detected with respect to pain intensity reduction and maximum mouth opening improvement.
